# High-Efficiency Photoinduced Charge Separation in Fe(III)carbene Thin Films

**DOI:** 10.1021/jacs.3c05404

**Published:** 2023-08-24

**Authors:** Minli Zhang, Catherine E. Johnson, Aleksandra Ilic, Jesper Schwarz, Malin B. Johansson, Reiner Lomoth

**Affiliations:** † Department of Chemistry - Ångström Laboratory, Uppsala University, Box 523, SE-75120 Uppsala, Sweden; ‡ Center for Analysis and Synthesis, Department of Chemistry, Lund University, Box 124, SE-22100 Lund, Sweden

## Abstract

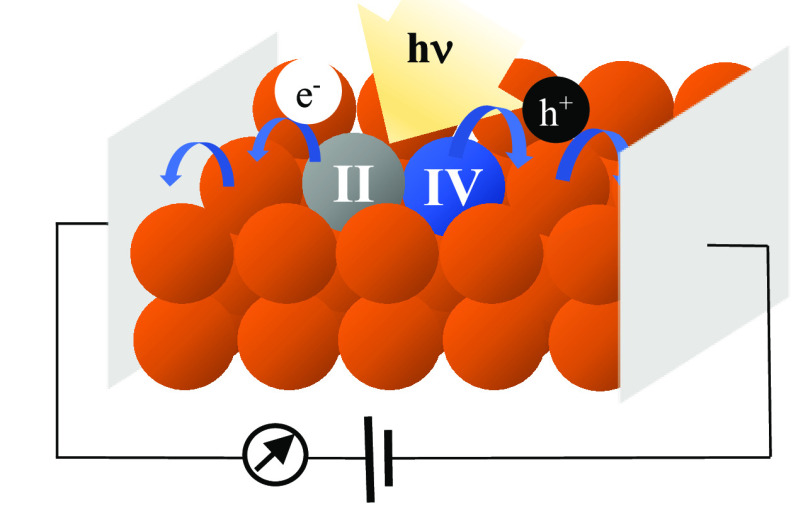

Symmetry-breaking charge separation in molecular materials has attracted increasing attention for optoelectronics based on single-material active layers. To this end, Fe(III) complexes with particularly electron-donating *N*-heterocyclic carbene ligands offer interesting properties with a ^2^LMCT excited state capable of oxidizing or reducing the complex in its ground state. In this Communication, we show that the corresponding symmetry-breaking charge separation occurs in amorphous films of pristine [Fe(III)L_2_]PF_6_ (L = [phenyl(tris(3-methylimidazol-2-ylidene))borate]^−^). Excitation of the solid material with visible light leads to ultrafast electron transfer quenching of the ^2^LMCT excited state, generating Fe(II) and Fe(IV) products with high efficiency. Sub-picosecond charge separation followed by recombination in about 1 ns could be monitored by transient absorption spectroscopy. Photoconductivity measurements of films deposited on microelectrode arrays demonstrated that photogenerated charge carriers can be collected at external contacts.

Synthetic molecular systems undergoing charge transfer from a photoexcited chromophore to an identical moiety in the ground state, thus breaking the symmetry of the chromophore pair, keep attracting significant interest.^1−7^ Inspired by the special pair in photosynthetic charge separation, much of this interest emerges from prospective applications for the conversion of radiant to electrical or chemical energy via photovoltaics^8^ or artificial photosynthesis.^9^ While symmetry-breaking charge separation (SBCS, for reviews see^10−13^) is rarely observed in bimolecular reactions,^14,15^ SBCS has been studied extensively in weakly coupled multichromophoric architectures controlling chromophore–chromophore interactions by covalent linking (bianthryl,^16−18^ assemblies of perylene,^7,19,20^ perylenediimide,^1−4,21−27^ and other dyes^5,28−32^) or in suitable scaffolds.^6,33^

The vast majority of these systems have been studied in fluid solutions, while solid materials could be more easily integrated into, *e.g*., organic photovoltaics or other devices where SBCS would enable light harvesting and charge separation in a single molecular material without the need for exciton migration to a donor–acceptor interface. Solid materials not only avoid practical problems with the integration of liquid components but might also enable efficient SBCS between unlinked chromophores due to their close proximity. However, the process is typically relying on solvation effects and/or conformational changes that favor charge separation over competing deactivation pathways.^5,7,22,31,34,35^ Cases of SBCS in the solid state are therefore scarce with few examples of perylene- and terylenediimides,^36−38^ cyanine,^39^ and copper phthalocyanine^40^ chromophores next to the chlorodipyrrin acceptor material used in organic photovoltaics.^8^ Systems with some intrinsic driving force not relying on solvation effects might therefore provide a promising approach to SBCS in solid materials. To this end, iron complexes with *N*-heterocyclic carbene (NHC) ligands, which have lately emerged as a novel class of photoactive compounds based on earth-abundant elements,^41−45^ offer interesting excited state reactivity.^46−49^ Specifically, it was recently demonstrated that the ligand-to-metal charge transfer (^2^LMCT) excited state of [Fe(III)L_2_]^+^ (L = [phenyl(tris(3-methylimidazol-2-ylidene))borate]^−^) undergoes SBCS in the solution phase.^15^ As the strongly electron-donating ligand places the Fe(IV/III) redox couple below the potential for ligand oxidation, the ^2^LMCT state with its π_L_^1^t_2g_^6^ electron configuration is correspondingly higher in energy than the Fe(II)+Fe(IV) charge separated state (t_2g_^6^ + t_2g_^4^; Scheme 1). The resulting intrinsic driving force for SBCS is an exceptional characteristic of this kind of chromophore, and modifications to the potentials of the metal- and ligand-based redox processes by ligand design might offer adjustable energetics of the SBCS process. It remained however to be demonstrated that the chromophore–chromophore interactions in solid FeNHC materials would be appropriate for efficient photoinduced electron transfer. Furthermore, it was unclear if the charge carriers would be sufficiently long-lived and mobile to enable charge transport through this kind of material.

**Scheme 1:**
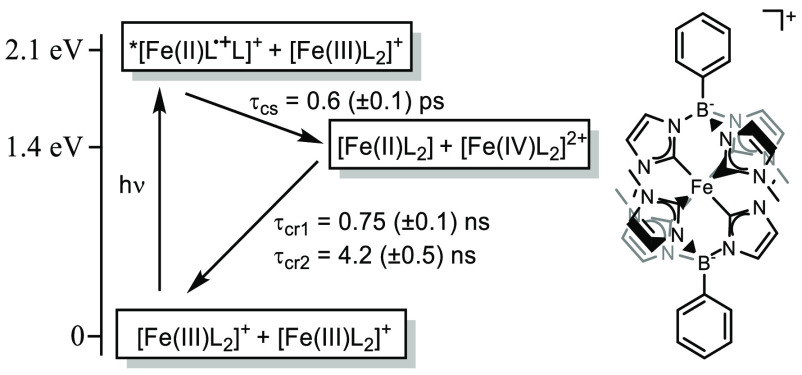
State Energy Diagram (left) Representing Symmetry Breaking Charge Separation (SBCS) and Charge Recombination (CR) upon Photoexcitation of [Fe(III)L_2_]^+^ and Molecular Structure of the Chromophore (right) Energies of the excited (^2^LMCT) and charge separated states are solution-based values (in acetonitrile).^15^ Time constants refer to the solid chromophore as a PF_6_^–^ salt.

Here, we report on a first example of photoinduced charge separation in a solid FeNHC material in the form of amorphous thin films of [Fe(III)L_2_]PF_6_. Ultrafast, highly efficient SBCS in these films was directly evidenced by transient absorption spectroscopy, which monitored the formation of electron–hole pairs, [Fe(II)L_2_] and [Fe(IV)L_2_]^2+^, from the ^2^LMCT excited state. Photoconductivity measurements further demonstrated that photogenerated charge carriers can be extracted from the film on external contacts or by redox reactions on the surface of the film.

Thin films of [Fe(III)L_2_]PF_6_ were deposited by drop casting on glass substrates coated with an array of 90 pairs of interdigitated gold microelectrodes (10/10 μm electrode/gap width; Figure 1a). The integrity of the complexes in the thin films was confirmed by Raman spectroscopy (Figure S1). Electron microscopy revealed that the central parts of the deposits placed on the active area of the IDE (3.5 mm diameter) form a compact film that completely covers the Au fingers and the gaps in between (Figure 1b). Micrographs further indicate that the film’s approximate thickness is comparable to that of the Au fingers (0.20 μm). Based on the absence of any X-ray diffraction pattern, the films investigated in this study can be described as essentially amorphous (Figure S10).

**Figure 1:**
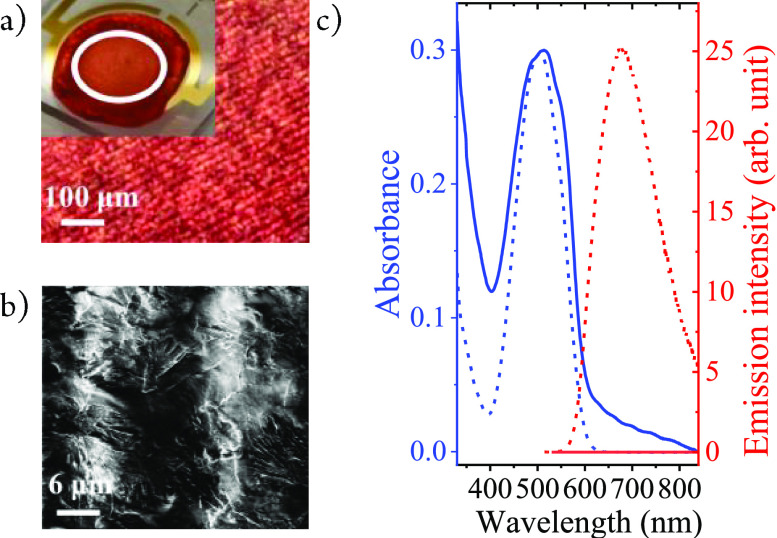
(a) Optical microscopic and (b) scanning electron microscopy (SEM) images of the [Fe(III)L_2_]PF_6_/IDE film. The inset is the picture of the IDE sample with the white circle indicating the illuminated area (2.5 mm diam). (c) Emission (red lines) and absorption spectra (blue lines) of [Fe(III)L_2_]PF_6_ in DMF solution (dashed lines) and as a solid film (solid lines).

The electronic absorption spectra of the films are very similar to the solution phase spectrum, with the visible absorption dominated by the LMCT absorption band peaking at about 500 nm (Figure 1c). On the IDE substrate there is minor additional absorption to the red that is not seen in solution. The tail starting from 600 nm does not agree with the spectra of either the Fe(II) or the Fe(IV) oxidation states^15,46,50^ and can be attributed to aggregation effects in agreement with the much broader, red extended absorption of the crystalline powder (Figure S8). Any contribution of degradation products could be ruled out by the good agreement between Raman spectra of the film and a powder sample of pristine [Fe(III)L_2_]PF_6_ (Figure S1). Emission spectra of the films demonstrate that the photoluminescence of the complex is entirely quenched in the solid state (Figure 1c). This is much more efficient than in the solution phase where not more than one-third of the excited [Fe(III)L_2_]^+^ could react on the picosecond time scale with a nearby complex, even at concentrations close to saturation (∼0.1 M).^15^ With 2% emission quantum yield in dilute solution, it can be estimated that this yield is reduced by at least 2 orders of magnitude in the film in order to drop below the detection limit. With an unquenched lifetime of 2 ns, this indicates efficient quenching in less than 20 ps, in agreement with the femtosecond-transient absorption data (see below).

To directly observe the excited state dynamics of solid [Fe(III)L_2_]PF_6_, films on the IDEs were studied by femtosecond transient absorption (TA) spectroscopy. After excitation with 500 nm pulses (∼150 fs, 1 mW/cm^2^), TA spectra initially show the formation of a pronounced absorption band peaking at about 360 nm that emerges within the first 300 fs, essentially within the instrument response (Figure 2a, blue spectra). This absorption band and the absence of any ground state bleach are characteristic of the ^2^LMCT excited state that can be observed in dilute solutions where it returns directly to the ground state with its intrinsic lifetime of 2 ns (Figure S2).^15,46^ In contrast to the dilute solution, the TA data of the film shows however no stimulated emission.^15,46^ Notably, the excited state in solid [Fe(III)L_2_]PF_6_ does not return directly to the ground state but evolves into a new state that is characterized by (i) a similar, slightly stronger absorption at 380 nm, (ii) a broader, much more intense absorption in the red, and (iii) a pronounced bleach of the ground state absorption with isosbestic points at about 445 and 575 nm (Figure 2a, red spectra). These features are in excellent agreement with the spectra of the different oxidation states in solution,^15,46,50^ and the expected differential absorption for the SBCS process (Figure 2a, Figure S3) nicely traces the TA spectra after the first picoseconds when the peak concentration of the charge separation products has been reached. The blue absorption due to the Fe(II) product is very similar to the corresponding feature of the LMCT state but increases further during the first picosecond due to a somewhat larger differential absorption for the Fe(II) product relative to its excited state precursor. Extinction coefficients for the 360 nm band of the LMCT excited state (*[Fe(II)L^+^L]^+^) and the [Fe(II)L_2_] product are similar in solution,^15^ and the transient absorption data therefore indicate that the formation of charge separation products proceeds with a yield at least close to unity. The charge separation kinetics can be observed conveniently in the region of the Fe(III) LMCT absorption band where the negligible net absorption of the excited state is replaced for the pronounced bleach characteristic of the charge separated state and at wavelengths above 600 nm, where the pronounced absorption of the Fe(IV) product emerges. While the TA data reveal no spectral or kinetic features that could be attributed to vibrational cooling, contributions from nonthermalized species cannot be excluded. All TA features decay to baseline within a few nanoseconds indicating complete recovery of the Fe(III) ground state by charge recombination (Figure 2b). Global fitting of a three-exponential model to the transient absorption data results in time constants of 0.6 (±0.1) ps for charge separation and 0.75 (±0.1) and 4.2 (±0.5) ns for the biphasic recombination (see the Supporting Information). Global fit results are shown for selected wavelengths in Figure 2c.

**Figure 2:**
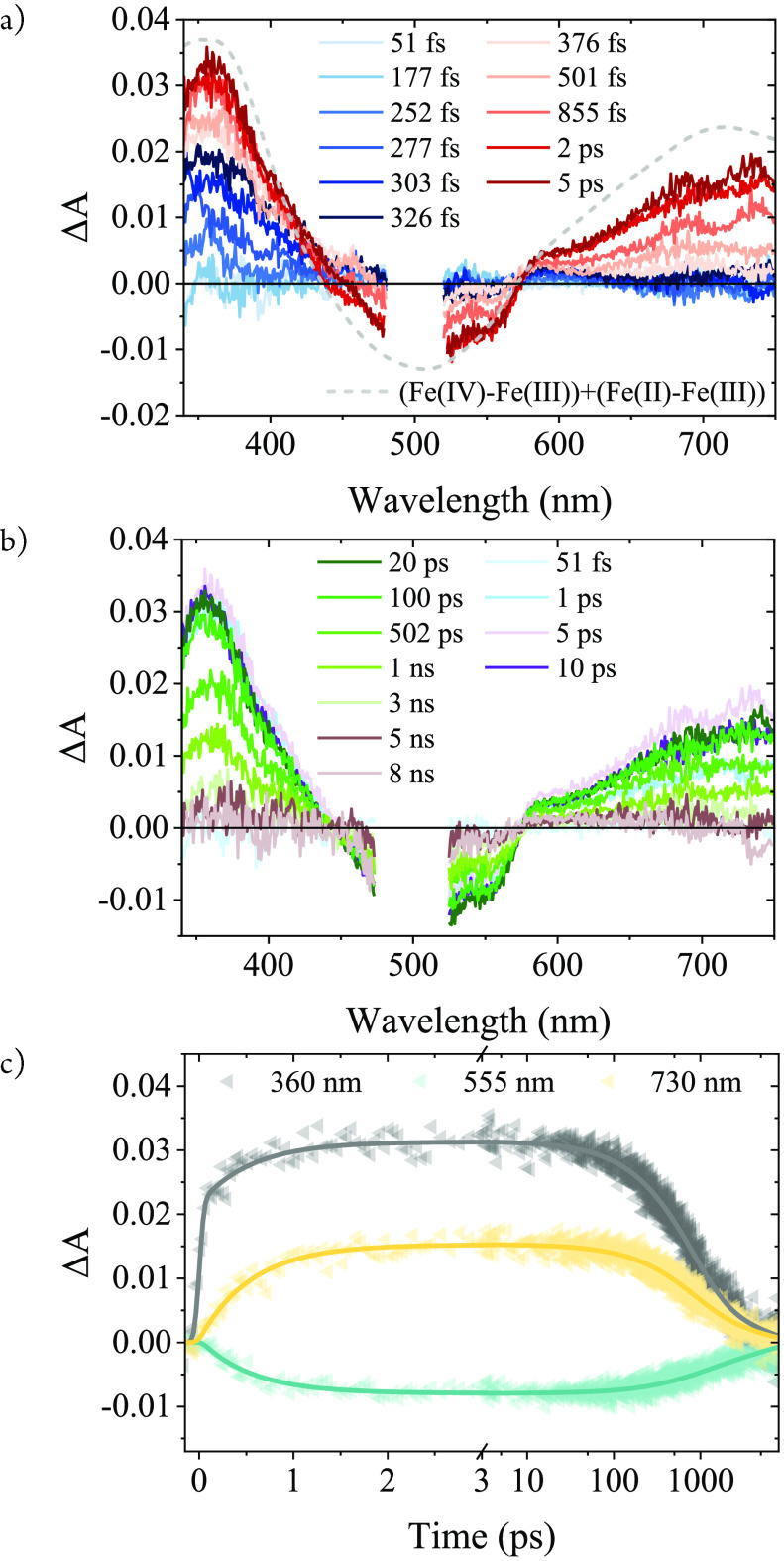
Selected fs-TA spectra of the [Fe(III)L_2_]PF_6_/IDE film measured at early (a) and late (b) times (λ_ex_ = 500 nm). The dashed line is the expected differential spectrum for the SBCS reaction (see Figure S3). (c) Kinetics at 360 nm (^2^LMCT excited state and Fe(II)), 550 nm (Fe(III) ground state), and 730 nm (Fe(IV)). Solid lines are fit results from global analysis.

In summary, the TA data provide unambiguous evidence that photoexcitation of [Fe(III)L_2_]^+^ in the solid state results in the same SBCS reaction previously observed in concentrated solution. The significantly exergonic (−0.7 eV) charge separation in solid [Fe(III)L_2_]PF_6_ is very fast (*k*_cs_ = 2 × 10^12^ s^–1^), similar to the reaction between pairs of [Fe(III)L_2_]^+^ in concentrated solution (6 × 10^11^ s^–1^),^15^ and significantly faster than the charge separation in perylenediimide films (6 × 10^10^ s^–1^).^36^ Importantly, the efficient quenching in the solid state can be attributed exclusively to the SBCS reaction based on the essentially quantitative formation of charge separated products, comparable to the quantitative or near quantitative charge separation in films of tetra(phenoxy)perylenediimide^36^ or pentamethine cyanine salts.^39^ Recombination in solid [Fe(III)L_2_]PF_6_ is however 30-fold slower in the solid state than geminate recombination in the solution phase^15^ that prevents the majority of products from escaping the solvent cage. This advantage of the solid state might be attributed to small reorganization energies of the electron transfer reactions that can be expected in the absence of a solvent contribution and with the minor structural differences between the oxidation states of FeNHC complexes (Fe(III)/Fe(IV)^46,50^ Fe(II)/Fe(III)^47,51^). As a result, the probably small reorganization energies could disfavor the relatively strongly exergonic (−1.4 eV) recombination (inverted region) while favoring charge transport by self-exchange.^52^

To investigate whether the photogenerated charge carriers are sufficiently mobile to be extracted on external contacts under an applied bias, we studied the photoconductivity of the films. I/V curves (Figure 3a) show a strongly enhanced current with almost ohmic behavior over a ±5 V range (ca. 2.2 × 10^–8^ Ω^–1^ cm^–1^ under white light illumination, 0.58 mW/cm^2^, see the Supporting Information). The photocurrent’s wavelength dependence (Figure 3b) traces the visible absorption of the [Fe(III)L_2_]PF_6_ film, confirming that the involved charge carriers originate from the reactivity of the lowest ^2^LMCT excited state. The dependence of the photocurrent on illumination power (see the Supporting Information) agrees with the expected limitations from recombination losses that increase at higher carrier concentrations. Nevertheless, we estimate that at a bias of 5 V about 0.8% of the carriers (per absorbed photons) can be collected at the electrodes (see the Supporting Information) despite the relatively large gap of 10 μm.

**Figure 3:**
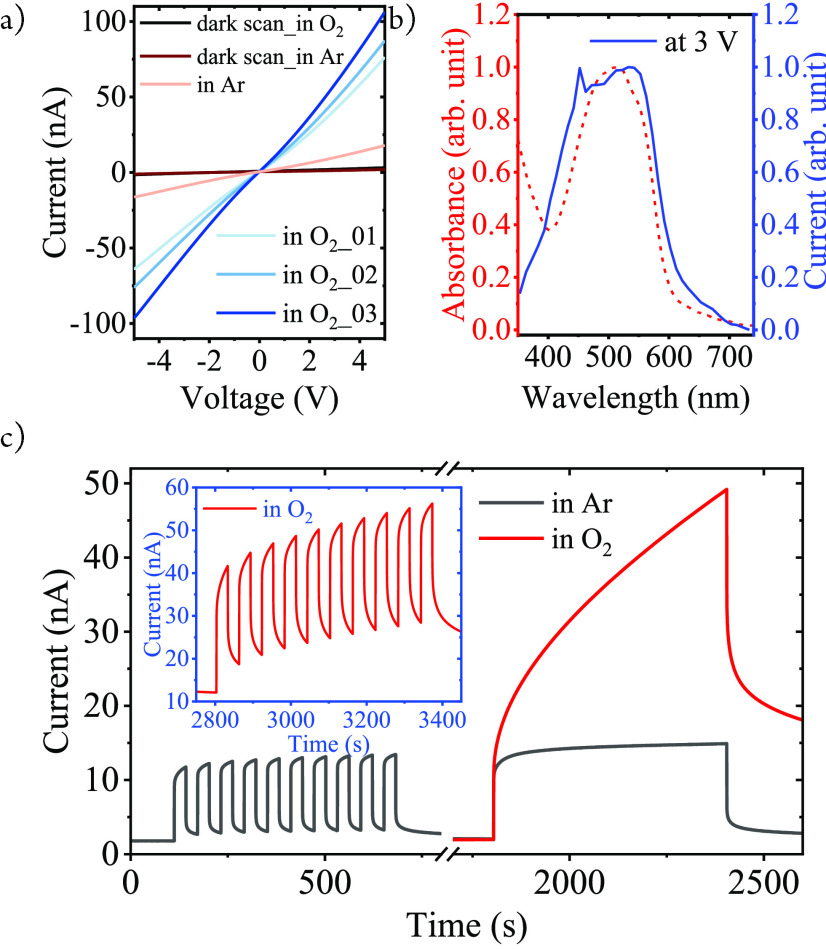
Photoconductivity of the [Fe(III)L_2_]PF_6_/IDE film (at room temperature). (a) I/V curves (white light, 0.58 mW/cm^2^) in Ar and O_2_ (scans 1–3). (b) Spectral response (Ar, 3 V bias). (c) Photoresponse in Ar and O_2_ (white light, 30s/30s on/off cycles).

Interestingly, the photocurrent response upon modulation of the illumination revealed two different time regimes (Figure 3c). A rapid, quasi instantaneous rise with the 100 ms time resolution of the recording and much slower components that are seen in the rise and drop of the photocurrent. Under an Ar atmosphere, the delayed rise and persistent photoconductivity are not very prominent, but corresponding effects are much more pronounced under O_2_. The Fe(II) state is known to be readily oxidized by atmospheric oxygen, and we tentatively attribute the effects of O_2_ to the scavenging of Fe(II) carriers at the surface of the film. Depleting the concentration of Fe(II) carriers near the surface should increase the lifetime of the Fe(IV) carriers and result in the delayed and persistent conductivity.^53^ Nevertheless, the persistent photoconductivity vanishes completely after corresponding resting times, and the behavior of the electrodes is fully reversible, showing no signs of degradation of the active material upon extended light/dark cycling with applied bias under either atmosphere as evidenced by the electronic absorption and Raman spectra of the films (Supporting Information).

In conclusion, we could establish that photoexcitation of amorphous thin films consisting of pristine [Fe(III)L_2_]PF_6_ leads to highly efficient exciton separation that causes marked photoconductivity of the films. These observations represent the first example of intrinsic photogeneration and transport of charge carriers in a transition metal complex in the solid state. Electron–hole recombination is favorably slow relative to ultrafast generation. Thus, [Fe(III)L_2_]PF_6_ represents a promising class of molecular materials for optoelectronic and photocatalytic applications based on charge separation in a single-material active layer. We are currently refining procedures for film deposition and characterization to control film morphology and its effect on the photophysical properties. Future directions involve refined architectures involving rectifying junctions as well as modifications for photocatalytic applications.

## Supporting Information Available

Experimental details, Raman spectra of the films, additional UV–vis absorption and emission spectra, as well as fs-TA and photoconductivity data (PDF)
