# Supplementary material for: High-Efficiency Photoinduced Charge Separation in Fe(III)carbene Thin Films
Source: J Am Chem Soc. 2023 Aug 24;145(35):19171–6. doi: 10.1021/jacs.3c05404 (PMC10485928; doi:10.1021/jacs.3c05404)
Supplement: Supplementary file 1 — ja3c05404_si_001.pdf [file ja3c05404_si_001.pdf]

Supporting Information

High-Efficiency Photoinduced Charge Separation in Fe(III)carbene Thin Films

Minli Zhang,[1] Catherine E. Johnson,[1] Aleksandra Ilic,[2] Jesper Schwarz,[2] Malin B. Johansson,[1] Reiner Lomoth[1]*

[1] Department of Chemistry - Ångström Laboratory, Uppsala University, Box 523, SE-75120 Uppsala, Sweden.

2 Center for Analysis and Synthesis, Department of Chemistry, Lund University, Box 124, SE-22100 Lund, Sweden

Table of Contents

Page	Content
S2	Experimental Methods
S4	Raman Spectroscopy
S5	Ultrafast Transient Absorption Spectroscopy
S7	UV-Vis-NIR Absorption Spectroscopy
S9	Scanning Electron Microscopy
S9	X-ray Diffraction
S10	Photoconductivity Characterization
S13	Reference

1. Experimental Methods

General Information

DMF (for spectroscopy Uvasol®) and acetonitrile (for spectroscopy Uvasol®) were obtained from Merck. All solvents were used as received without further purification. Interdigitated electrode (IDE) substrates with 90 pairs of Au electrode fingers (10 µm electrode width, 10 µm electrode gaps, and 200 nm (Ti/Au 50/150 nm) electrode thickness) were obtained from MicruX Technologies.

All experiments were performed at room temperature unless stated otherwise.

Film Preparation

Sample $[Fe(III)L_2]PF_6$ was dissolved in DMF at a concentration of 20 mg mL^{-1}. 2 µL $[Fe(III)L_2]PF_6$ complex solution was drop cast onto the interdigitated electrode substrate and then the substrate was moved to a vacuum chamber. To remove the solvent, the substrate was kept at -1.0 bar for 2 h and then heated on a 100 °C hot plate for 10 min.

Scanning Electron Microscopy

Scanning electron microscopy (SEM) images were taken using an LEO 1530 FEG instrument (LEO Electron Microscopy Ltd., Cambridge, UK) with an in-lens detector operating at 5.00 kV and 3.05 kV.

Raman Spectroscopy

Raman spectra were acquired using a Renishaw inVia Raman confocal spectrometer equipped with 50x objective from Leica. A 785 nm diode laser (maximum power of 500 mW) in the edge mode was used as the light source for collecting spectra, and 1200 lines/mm gratings were used as the dispersive element. The focus size is about 25 µm by 5 µm.

X-ray Diffraction

X-ray diffraction (XRD) pattern was recorded using a Bragg-Brentano set-up (Siemens D5000 Th-Th) with Cu Kα (λ = 1.54051 Å) radiation.

Absorption and Emission spectra

The absorption spectra were collected with a Varian Cary5000 spectrophotometer. The emission spectra were recorded using Edinburgh Instrument FLS1000, all samples were excited at 500 nm. All samples were background subtracted and corrections for the wavelength-dependent instrument response were made for emission spectra.

Transmittance and reflectance measurements were performed on a Perkin-Elmer Lambda 900 double-beam UV/vis/NIR spectrophotometer, which was equipped with an integrating sphere attachment, using a Spectralon reflectance standard.

Femtosecond Transient Absorption Spectroscopy

Femtosecond-TA spectra were measured on a Newport TAS system with Coherent Libra Ti:sapphire amplifier (800 nm, 3 kHz, 1.5 mJ, fwhm 40 fs). The excitation wavelength (500 nm) was generated by the optical parametric amplifiers (TOPAS-C, Light Conversion). A CaF_2 crystal was used to generate the white supercontinuum probe light. Pump-probe overlap was optimized at the sample, and the pump power was adjusted to ca. 3 mW for the solution and 1 mW for the film. IDE film sample was measured

under ambient conditions. Spectra chirp correction and data fitting (using global analysis) were performed with Surface Xplorer v.4 and Glotaran 1.5.1 software,[1] respectively.

The pump power P was measured to be 1 mW, with a repetition rate R of 3 kHz (where every second pump pulse was blocked by the chopper). Hence, the energy per pulse, E_{pulse}, can be calculated as:

$$E_{pulse} = \frac{P}{R} = \frac{1\,mW}{1500\,s^{-1}} = 667nJ$$

The area of the pump pulse, A_{pump}, was determined by the use of a beam profiler which measured the major and minor diameters of the ellipse-shaped pulse (d_1 = 203.2 μm, d_2 = 206.8 μm), and gave a value of:

$$A_{pump} = \pi r_1 r_2 = \pi\left(\frac{0.02032}{2}\right)\left(\frac{0.02068}{2}\right)cm^2 = 3.30 \times 10^{-4}\,cm^2$$

The pump fluence, F_{pump}, which is the average energy per pulse per area, is therefore given by:

$$F_{pump} = \frac{E_{pulse}}{A_{pump}} = \frac{667\,nJ}{3.30 \times 10^{-4}\,cm^2} = 2.0\,mJ\,cm^{-2}$$

With a photon energy of $3.97 \times 10^{-19}\,J$ (500 nm) the photon fluence F_{photon}, is given by:

$$F_{photon} = \frac{F_{pump}}{E_{photon}} = \frac{2.0\,mJ\,cm^{-2}}{3.97 \times 10^{-19}\,J} = 5.1 \times 10^{15}\,cm^{-2}$$

Films with an absorptance ($A = 1 - 10^{-Abs}$) of about 0.4 therefore absorbed about 2×10^{15} photons per pulse per cm^2. With a unit cell volume of the complex (2253.7 $Å^3$ [2]) and an approximate film thickness of 0.2 μm we estimate that the films contain about 1×10^{16} complexes per cm^2.

Photoconductivity

All photoconductivity measurements were conducted in Ar or O_2 atmosphere with a blue LED (LUXDRIVE, 443 nm, with BUCKPUCK 3023 LED driver, adjustable output power) or a white light LED lamp (Everlight, 17 W, 5000 K, 0.58 mW cm^{-2} at sample site) as the light source. Light exposure was controlled with a mechanical shutter (UniBlitz 225L2A0T5 controlled by a UniBlitz T132 Shutter Driver, < 10 ms opening/closing time). Photocurrents were recorded using an IviumStat potentiostat by applying bias voltage from -5 V to 5 V, the scan rate was 50 mV s^{-1}. A mask with a 2.5 mm diameter round hole was used to cover the IDE sample, leaving an active area of ca. 0.049 cm^2 for the interdigitated electrode substrate. The power of the white lamp was detected by an Ophir Photonics NOVA II power meter with a detection area of 1.2 cm × 1.2 cm, the power of the blue LED (443 nm) was detected by the same power meter but using a 2.5 mm diameter mask to confine the active area.

Wavelength-dependent measurements were conducted in a system consisting of a Xenon lamp and monochromator (Spectral products ASB-XE-175 and CM110).

Power-dependent measurements were conducted by adjusting the electric input power for the blue LED, the output light power that reached the IDE sample was detected by an Ophir Photonics NOVA II power meter.

2. Raman Spectroscopy

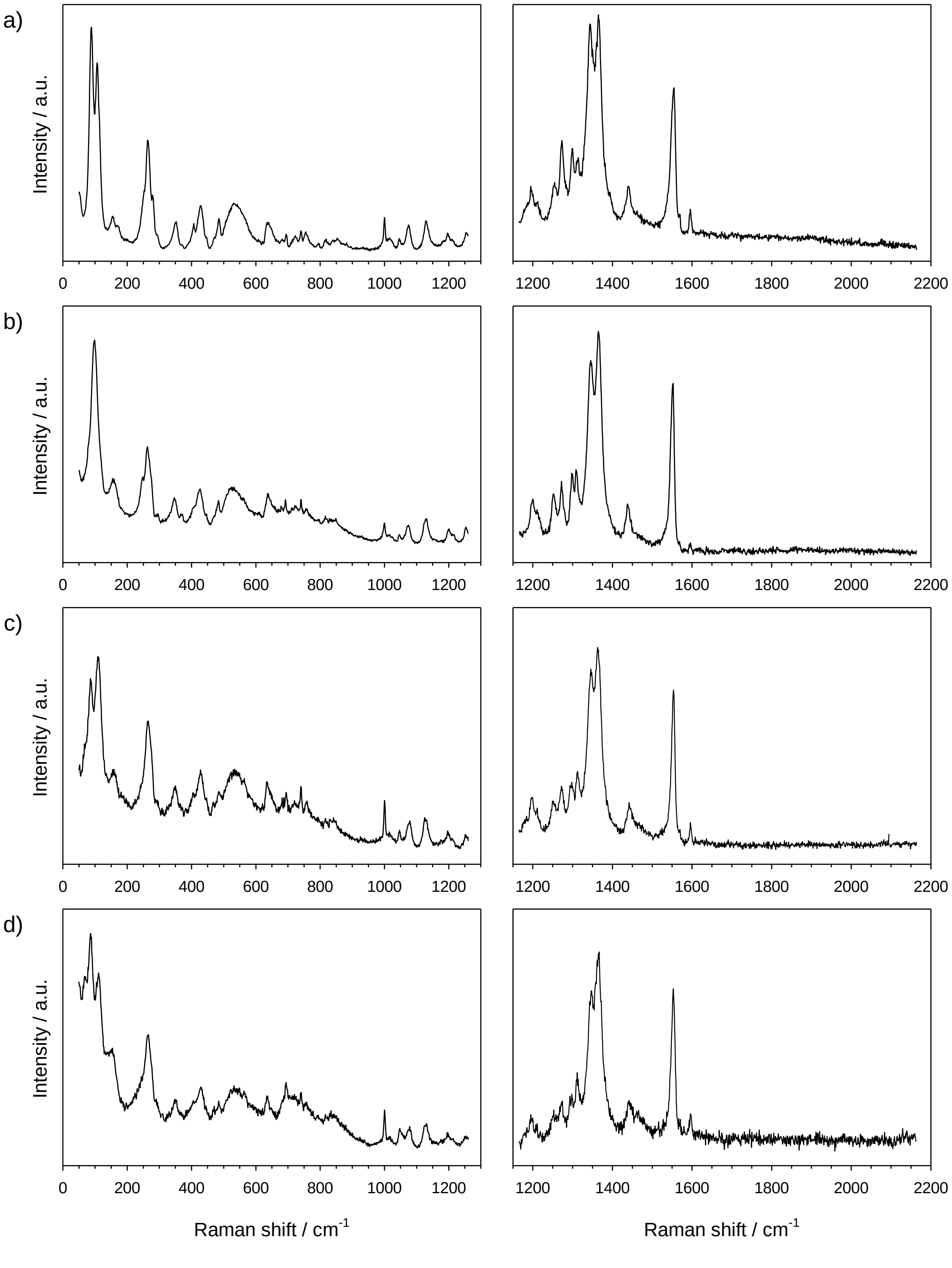

Figure S1. Raman spectra (λ_{in} = 785 nm) of solid [Fe(III)L_2]PF_6. a) Powder between quartz plates. b) Thin film drop-casted on IDE substrate. c) Thin film drop-casted on IDE substrate after annealing at 100 °C for 10 min. d) Thin film on IDE substrate (drop-casted, annealed at 100 °C for 10 min) after fs-TA spectroscopy and photoconductivity (Ar, O_2) measurements.

3. Ultrafast Transient Absorption Spectroscopy

Transient Absorption Spectra in Solution

The transient absorption spectrum of the ^{2}LMCT excited state (next to some stimulated emission) can be observed in fs-TA experiments with a dilute solution where contributions of the SBCS reaction to the excited state decay are negligible.

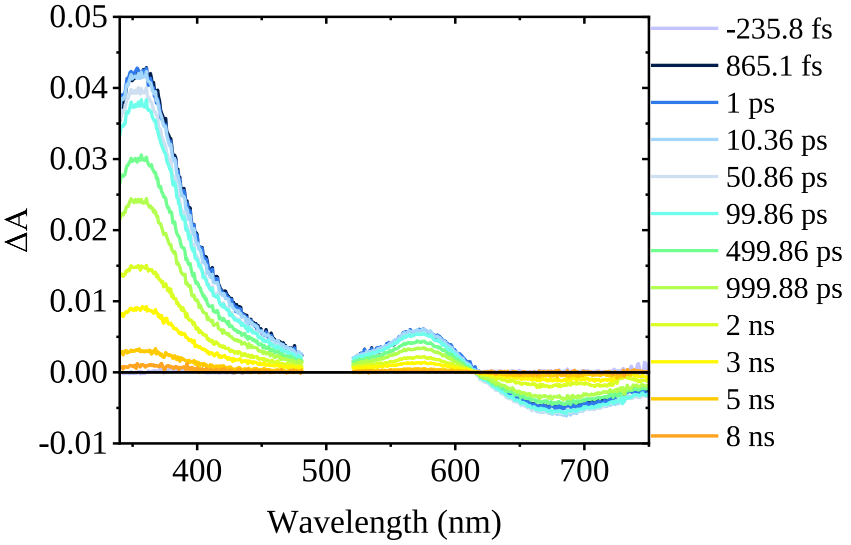

Figure S2. Selected fs-TA spectra of dilute $[Fe(III)L_2]PF_6$ acetonitrile solution, λ_{ex} = 500 nm; power = ca. 3 mW; absorbance = 0.15 at the excitation wavelength; pathlength = 1 mm.

Expected Differential Spectrum for SBCS Reaction

The differential spectrum for the formation of the charge separated state can be obtained from spectra of the diferent oxidation states that were determined by spectroelectrochemistry.[2]

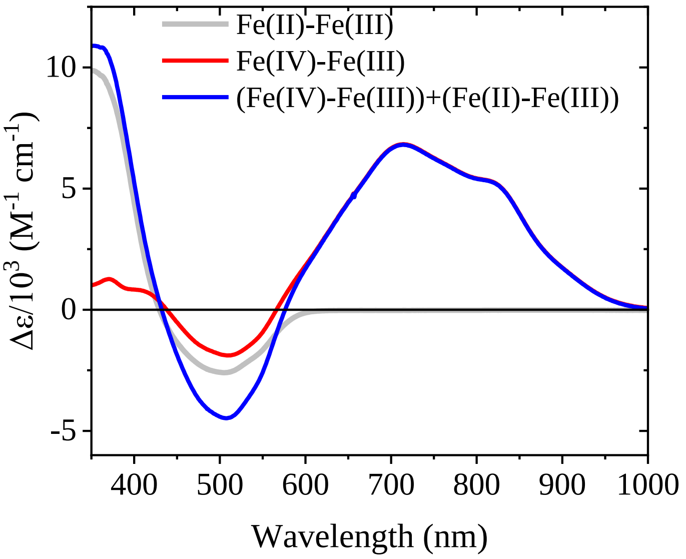

Figure S3. Differential spectrum of the SBCS reaction based on the absorption spectra of $[Fe(II)L_2]$, $[Fe(III)L_2]^+$ and $[Fe(IV)L_2]^{2+}$ in acetonitrile solution.[2]

Global Analysis

Before fitting, pre-zero signal was subtracted from all data to reduce excitation scatter. To a first approximation, the data can be described with two exponential terms describing the decay of the excited and the recombination of the charge separated state (Fig. S4). More accurate fitting requires an additional exponential term (Fig. S5).

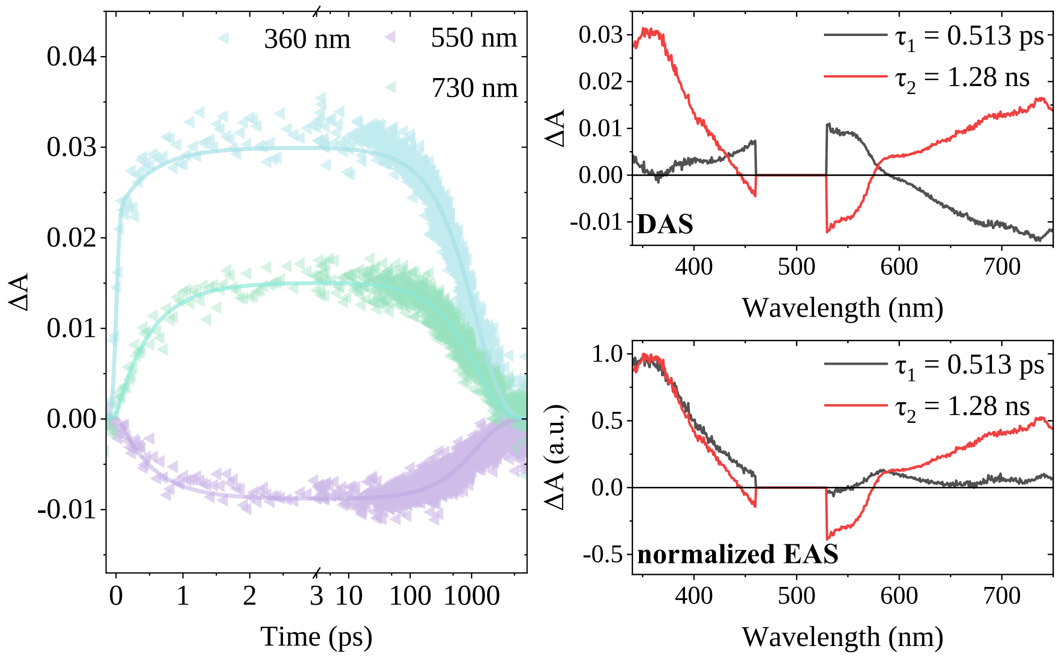

Figure S4. Results from global analysis with a biexponential model.

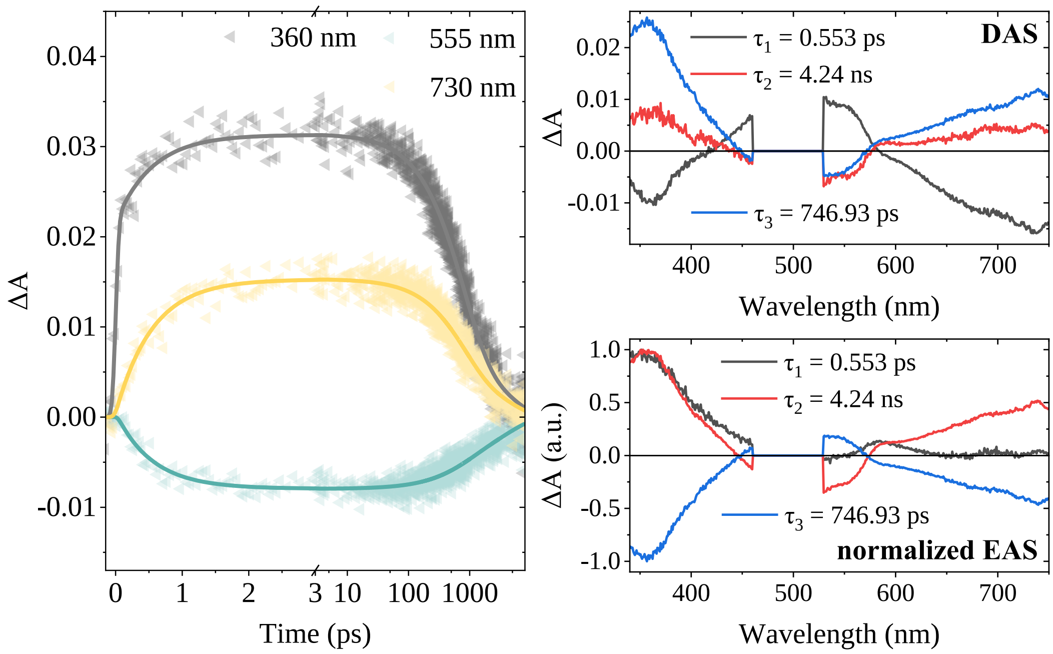

Figure S5. Results from global analysis with a three-exponential model.

4. UV-Vis-NIR Absorption Spectroscopy

Absorption spectra of films of [Fe(III)L_2]PF_6 on the IDE substrates were recorded in transmission mode as the necessary masking (exposing only the central 2.5 mm with a homogeneous coating) did not allow us to collect satisfactory spectra with the integrating sphere. Spectra of the powder sample and the larger films on quartz slides were recorded with an integrating sphere that allowed to collect transmittance and reflectance spectra and calculate the absorptance spectra of these samples. While the spectra of the films on IDE substrates might be affected by the scattering and reflective nature of the sample, the broadening of the LMCT band and the presence of a low-energy tail is unambiguously confirmed by the spectra of films on quartz slides and of the powder (Fig. S8). Within the precision of the measurements, the absorption spectra of the films also don't change upon annealing (Fig. S6) and after the intense illumination during transient absorption measurements and photoconductivity measurements (Fig. S7).

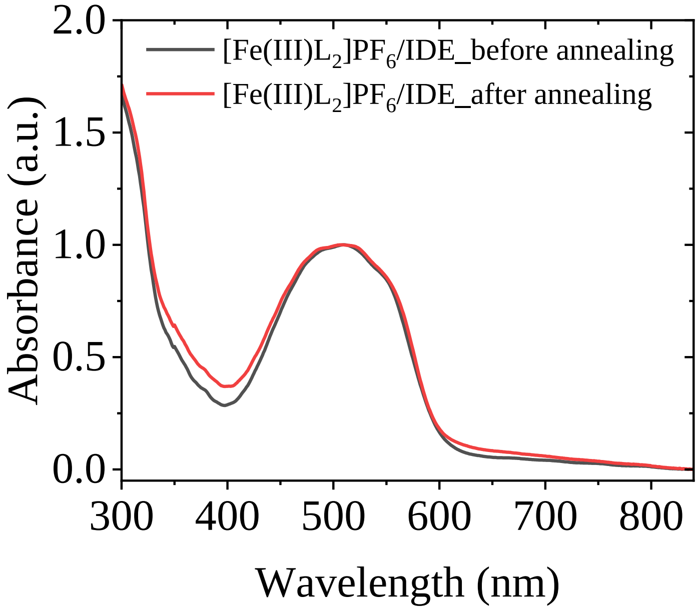

Figure S6. Scaled absorption spectra of [Fe(III)L_2]PF_6 film on IDE substrate before and after annealing at 100 °C for 10 min.

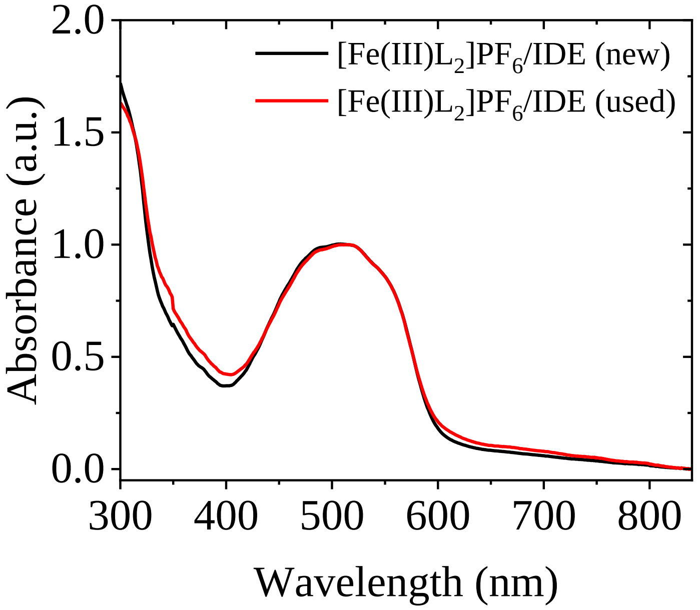

Figure S7. Scaled absorption spectra of freshly-made $[Fe(III)L_2]PF_6$ film on IDE substrate(drop-casted, annealed at 100 °C for 10 min) (black line), and $[Fe(III)L_2]PF_6$ film on IDE substrate (drop-casted, annealed at 100 °C for 10 min) after fs-TA spectroscopy and photoconductivity (Ar, O_2) measurements (red line).

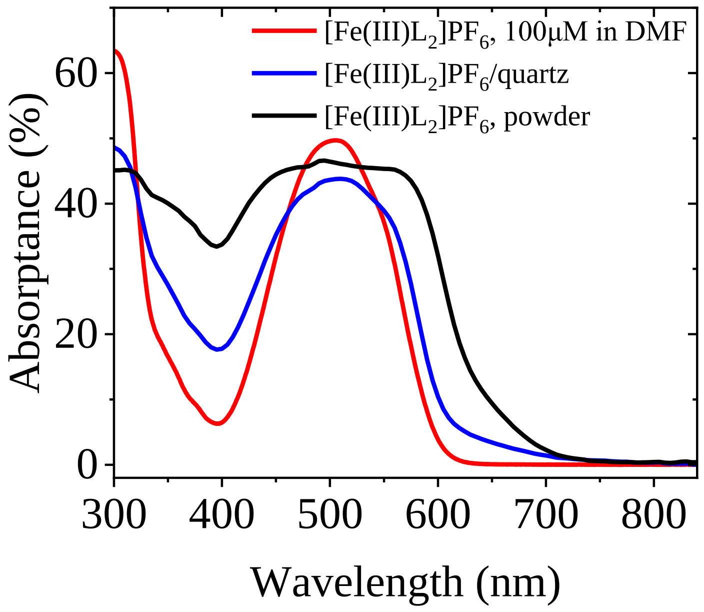

Figure S8. Absorptance spectra of $[Fe(III)L_2]PF_6$ in DMF solution, as thin films on quartz (prepared by drop casting and annealing) and as powder between quartz slides. Absorptance ($A = 1 - R - T$) of the powder and the thin film was determined from measurements of transmittance (T) and reflectance (R) in an integrating sphere. Absorptance of DMF solution was calculated from the measured absorbance as $A = 1 - 10^{-Abs}$.

5. Scanning Electron Microscopy

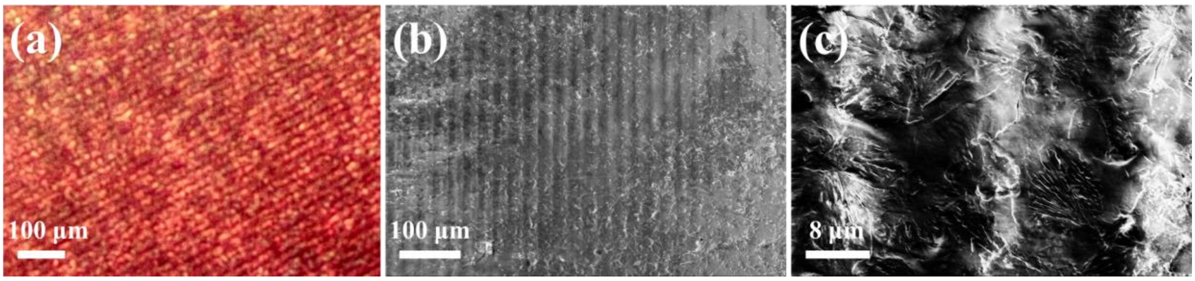

Figure S9. Optical microscopic image (a) and scanning electron microscopy (SEM) images of the [Fe(III)L_2]PF_6/IDE film (b, c). (a) and (c) is the same as the figures shown in the main text.

The red color in the microscopic image is from the [Fe(III)L_2]PF_6 sample, which shows that the whole interdigitated electrode working area is completely covered by [Fe(III)L_2]PF_6. The yellow lines in the picture are where gold electrode fingers are located, which can be seen easily under microscopy because the deposited very thin layer of [Fe(III)L_2]PF_6 makes the whole [Fe(III)L_2]PF_6 film semitransparent. SEM images in Figure S9b and Figure S9c show the morphology of the center part of the IDE sample with different magnifications. It can be seen that [Fe(III)L_2]PF_6 is deposited evenly on the whole IDE substrate. The relatively dark area in Figure S9c is the gap in between electrode fingers.

6. X-ray Diffraction (XRD)

The figure below shows the XRD pattern of the [Fe(III)L_2]PF_6 film deposited on the IDE substrate. No obvious diffraction peaks can be observed on the [Fe(III)L_2]PF_6/IDE film. Since Cu Kα radiation leads to the Fe characteristic fluorescent radiation which causes high background for the measurement, the refined XRD studies of [Fe(III)L_2]PF_6 films will require other source material and optimized configuration.

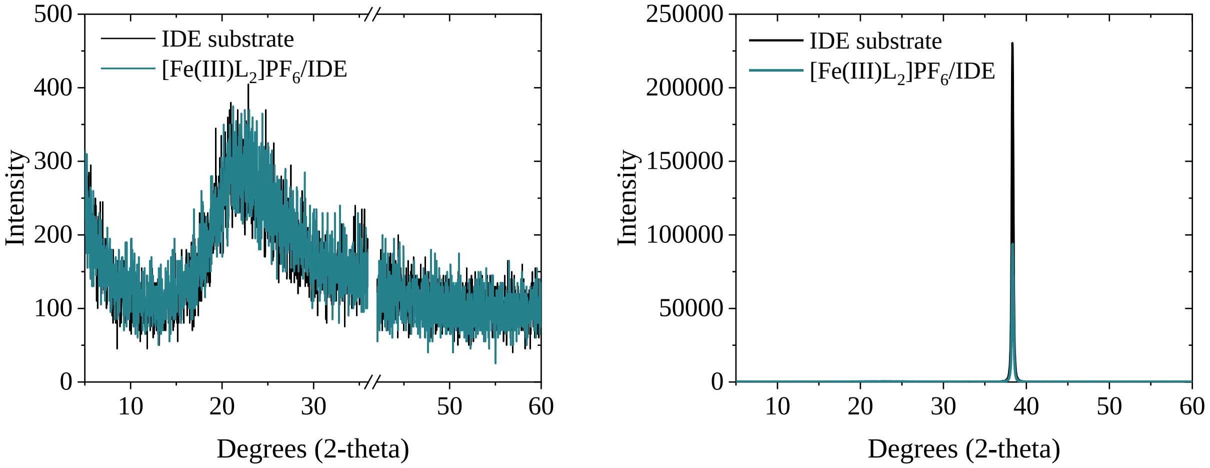

Figure S10. Left: XRD pattern of the [Fe(III)L_2]PF_6 film on the IDE substrate. The break on the x-axis is where the diffraction peak of the gold electrodes, the (111) plane at 38.2 2θ, is located. Right: the full XRD pattern that includes the diffraction peak of the gold electrodes.

7. Photoconductivity

Conductivity Calculation

The electrical conductivity (σ) of the [Fe(III)L_2]PF_6/IDE film is given by RA / l, where R is the resistance of the sample, and l and A are the distance between the electrodes and the cross-sectional area of the electrodes.

In a first approximation, the conductivity can be attributed to the material deposited in between the Au fingers. Thus, for the [Fe(III)L_2]PF_6/IDE architecture, l is considered as the width of the electrode finger gaps (10 μm). The total cross-sectional area can be estimated by multiplying the electrode finger thickness (0.2 μm) by the total finger length of ca. 80 mm (in the illumination area, 2.5 mm diameter). Accordingly, the current measured in the Ar atmosphere at 5 V bias voltage under white light illumination (0.58 mW cm^{-2}) is 17.93 nA, which corresponds to a conductivity of 2.2×10^{-8} Ohm^{-1} cm^{-1}.

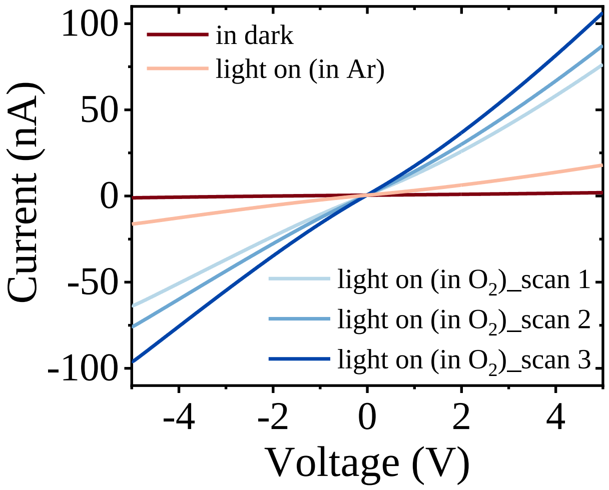

Figure S11. Optoelectronic response of the [Fe(III)L_2]PF_6/IDE film measured in the dark and under a white lamp exposure (0.58 mW/cm^2). The red line is the current curve measured in the Ar atmosphere, and the blue lines are the current curves measured in the O_2 atmosphere.

Power-dependence

The power dependence of the photoconductivity was studied with a dimmable LED (443 nm) (Figure S12 and Figure S13). The generated currents depend linearly on the illumination power up to about 0.06 mW cm^{-2} (ca. 3 μW, 0.049 cm^2).

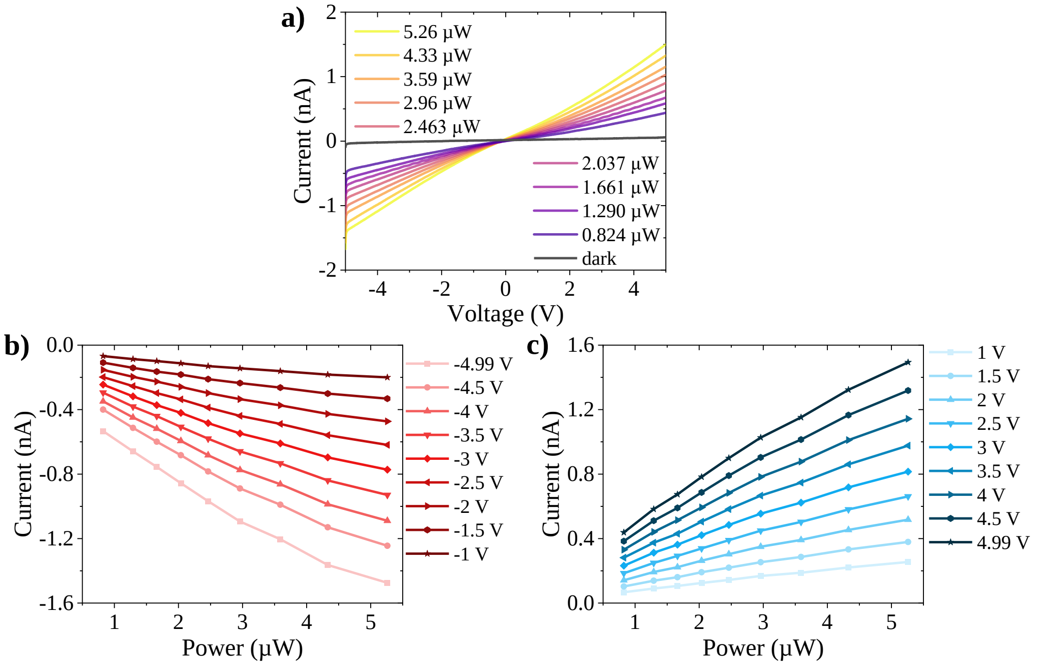

Figure S12. Power-dependent photoconductivity of the [Fe(III)L_2]PF_6/IDE film measured under lower illumination power density. (a) Current as a function of voltage in the +/-5 V range. (b) and (c) Current as a function of light power at different bias voltages. (Light power in the graphs is the illumination power of the blue LED that is recorded by a power meter through the same mask (2.5 mm diameter) used for the photoconductivity measurements.)

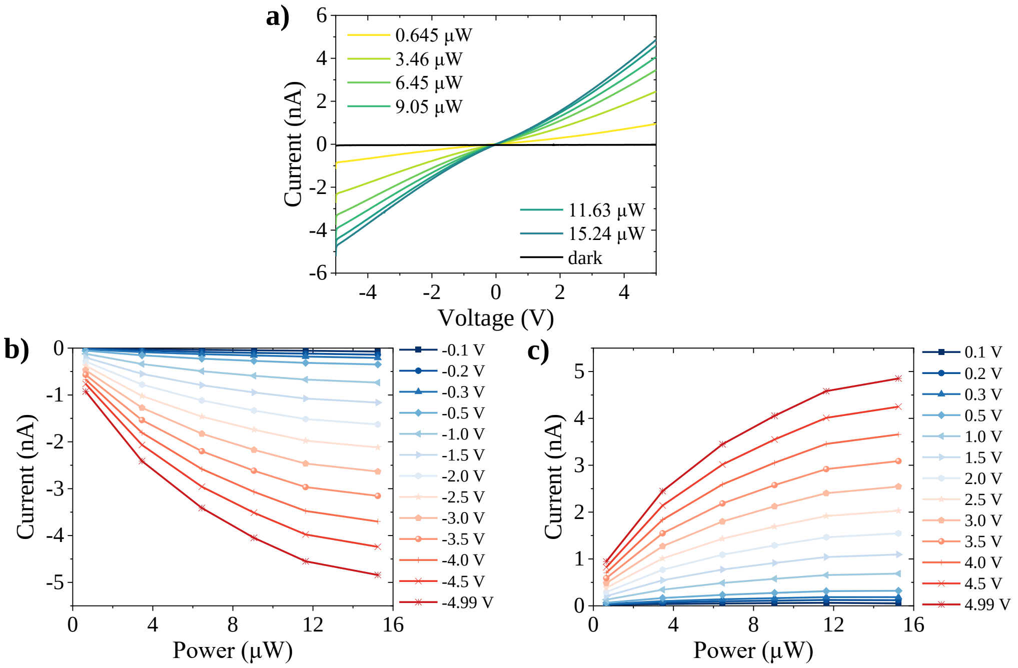

Figure S13. Power-dependent photoconductivity of the [Fe(III)L_2]PF_6/IDE film measured under higher illumination power density. (a) Current as a function of voltage in the +/-5 V range. (b) and (c) Current as a function of light power at different bias voltages. (Light power in the graphs is the illumination power of the blue LED that is recorded by a power meter through the same mask (2.5 mm diameter) used for the photoconductivity measurements.)

Charge Collection Efficiency

The charge collection efficiency (CCE) can be estimated according to the equation below:

$$CCE = \frac{I_{ph}\,dt}{N_{ph}(1-10^{-Abs})e}$$

where I_{ph} is the photocurrent (difference of current measured in the dark and under illumination), dt represents the duration for collecting the photocurrent and e is the elementary charge. Abs is the absorbance of the $[Fe(III)L_2]PF_6$ film at the excitation wavelength. N_{ph} is the number of the incident photons that reach the actual active area, which can be calculated by the equation below:

$$N_{ph} = \frac{P_{in}A_{spot}\,dt}{E_{photon}}$$

P_{in} is the power of the incident light, dt represents the duration of the measurement, and A_{spot} is the active working area. E_{photon} is the energy of per photon, which can be obtained from $E_{photon} = hc/\lambda$. For the IDE substrate, the working area is a 3.5 mm diameter circle where 90 pairs of 10 µm width Au electrodes are distributed at an even distance (10 µm) from each other, causing 50 % of the incident light to be blocked by the Au electrodes. Thus, the actual active working area of the $[Fe(III)L_2]PF_6$/IDE film should be $A_{actual} = 0.5 \times A_{spot}$, giving the number of photons falling on the area between the Au electrodes to be $N_{ph\_actual} = 0.5 \times N_{ph}$.

Taking the photocurrent generated in the Ar atmosphere at a 5 V bias voltage under the 0.824 µW blue LED illumination to be 0.38 nA, and the absorbance of $[Fe(III)L_2]PF_6$/IDE film at 443 nm to be ca. 0.17, the charge collection efficiency for $[Fe(III)L_2]PF_6$/IDE sample is 0.76%.

Optoelectronic Response under Ar Atmosphere and Blue LED Illumination

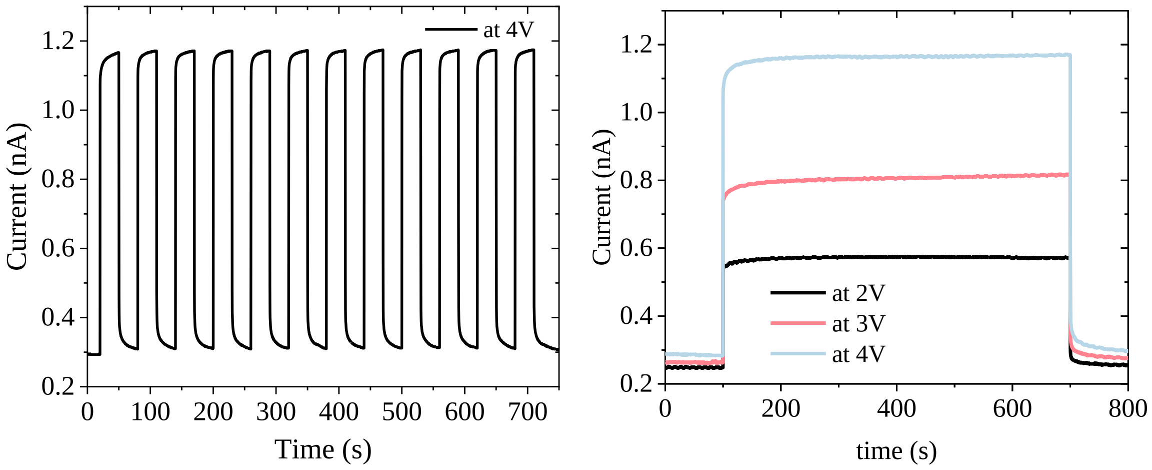

Figure S14. Left: Optoelectronic response of the $[Fe(III)L_2]PF_6$/IDE film measured (at a fixed bias voltage V = 4 V) in the Ar atmosphere under a blue LED illumination (0.3 mW cm^{-2}), with switching on/off duration of 20s/30s; Right: Bias voltage dependence of the current generated in a time frame of 600 s in the $[Fe(III)L_2]PF_6$/IDE film.

Reference

1. Snellenburg, J. J.; Laptenok, S.; Seger, R.; Mullen, K. M.; van Stokkum, I. H. M. Glotaran: A Java-Based Graphical User Interface for the R Package TIMP. *Journal of Statistical Software* **2012**, *49* (3), 1-22. https://doi.org/10.18637/jss.v049.i03.

2. Kjmer, K. S.; Kaul, N.; Prakash, O.; Chabera, P.; Rosemann, N. W.; Honarfar, A.; Gordivska, O.; Fredin, L. A.; Bergquist, K. E.; Haggstrom, L.; et al. Luminescence and Reactivity of a Charge-Transfer Excited Iron Complex with Nanosecond Lifetime. *Science* **2019**, *363* (6424), 249-253. https://doi.org/10.1126/science.aau7160.
